# SOX9, a potential tumor suppressor in cervical cancer, transactivates p21^WAF1/CIP1^ and suppresses cervical tumor growth

**DOI:** 10.18632/oncotarget.4133

**Published:** 2015-05-16

**Authors:** Hai-yan Wang, Ping Lian, Peng-Sheng Zheng

**Affiliations:** ^1^ Department of Reproductive Medicine, The First Affiliated Hospital, Xi'an Jiaotong University Medical School, Xi'an, China

**Keywords:** SOX9, cervical carcinoma, tumor suppressor, oncogene, p21^WAF1/CIP1^

## Abstract

Sex-determining region Y-box 9 protein (SOX9) is a transcription factor that may act as both oncogene and tumor suppressor depending on tumor origin. Here we found that SOX9 expression was progressively decreased in cervical carcinoma *in situ* and especially in invasive cervical carcinoma, compared with normal cervix tissue. The effects of SOX9 on the proliferation, viability, and tumor formation of cervical carcinoma cells were assessed through the silencing and overexpression of SOX9. Overexpression of SOX9 in cervical carcinoma cells (SiHa and C33A) inhibited cell growth *in vitro* and tumor formation *in vivo*. In agreement, the silencing of SOX9 in HeLa cells promoted cell growth in culture and tumor formation in mice. Overexpression of SOX9 transactivated p21^WAF1/CIP1^ via a specific promoter region, thus blocking G1/S transition. The quantitative chromatin immunoprecipitation analysis revealed physical interaction between SOX9 and the specific region of the p21^WAF1/CIP1^ promoter. We suggest that SOX9 is a potential therapeutic target in cervical carcinoma, that specifically transactivates p21^WAF1/CIP1^.

## INTRODUCTION

Cervical carcinoma is a common cancer with complex development and progression [1-7]. SOX9 is a stem cell-related transcription factor that functions in lineage restriction, terminal differentiation, and maintenance of stem cell properties [8]. Moreover, SOX9 plays an important role in multiple developmental programs, and thus its levels need to be strictly controlled in order for normal embryogenesis to occur [9]. It has been shown that SOX9 is overexpressed in cancers of the skin, prostate, lung, breast and brain, and contributes to tumor growth and invasion [10-14]. However, SOX9 serves as a tumor suppressor in some melanomas and endometrial carcinoma cells [15-17]. So the role of SOX9 in tumors is tissue-dependent. Although methylation of the SOX9 gene was found in cervical carcinogenesis [18], the role of SOX9 in cervical carcinoma remains unclear. In the present study, we found that SOX9 was down-regulated in the development and progression of human cervical tumor tissues. Furthermore we demonstrated that SOX9 inhibited cervical cancer cell proliferation and tumor growth, associated with transcriptional induction of p21^WAF1/CIP1^ (p21) by direct binding to a specific region of the p21 promoter.

## RESULTS

### SOX9 expression in cervical carcinogenesis

**Figure 1:**
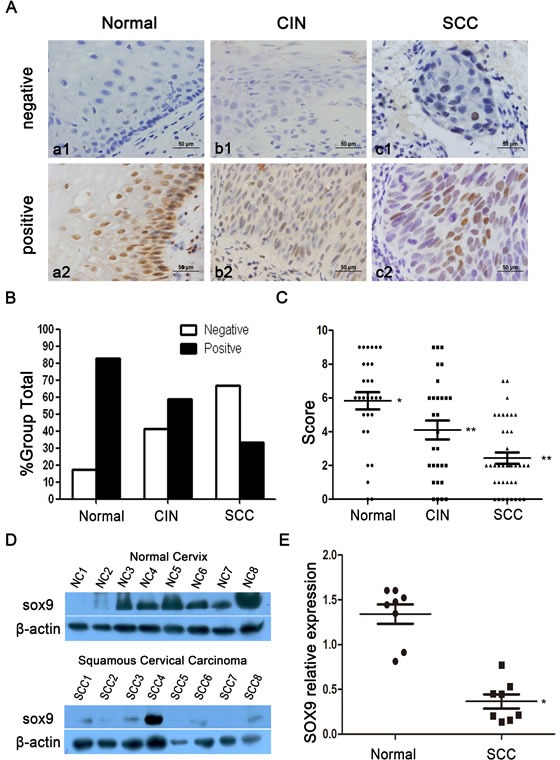
The expression of SOX9 is shown in normal cervical tissues and in tissues from different types of cervical lesions Images of immunohistochemistry (IHC) for SOX9 expression are shown in the normal cervix (a1-a2), cervical carcinoma *in situ* (b1-b2), and cervical carcinoma (c1-c2). Original magnifications, ×400. (**B**) SOX9 staining in 29 normal cervical tissues, 29 carcinoma *in situ* specimens, and 39 invasive carcinoma specimens is classified into negative and positive groups, and the percentages of positive cases and negative cases in each group are shown(The results of data comparison are showed in Table 1). (**C**) SOX9 expression in the normal cervix, carcinoma *in situ*, and cervical carcinoma is shown by scatter plot; the points represent the staining score for each specimen. (*t*-test, normal cervix vs. carcinoma *in situ*, *P < 0 .05; normal cervix vs. cervical carcinoma,** *P* < 0.01; carcinoma *in situ* vs. cervical carcinoma,** *P* < 0.01) (**D**) A Western blot shows the expression of SOX9 protein in the normal cervix and in squamous cervical carcinoma. (**E**) The staining density of the SOX9/β-actin ratio in each normal cervical specimen and squamous cervical carcinoma specimen was scanned by Alpha View SA software (*t*-test, *P < 0.05). Data shown are the mean±SD of triplicates.*P < 0 .05. **P < 0 .01.

To determine the protein expression of SOX9 in human cervical carcinogenesis, paraffin sections of normal cervical tissues and cancerous lesions were stained by immunohistochemistry. All SOX9 staining was observed in the nuclei of the positive cells in all cervical tissues, irrespective of whether the tissues were normal or cancerous (Fig. 1A). The percentage of specimens that stained positive decreased from 82.8% in normal cervical tissues to 58.6% in tissues representative of cervical carcinoma *in situ*, and ultimately to 33.3% in cervical invasive carcinoma tissues (Table 1 and Fig. 1B). The scatter plot reflects the IHC score of SOX9 staining (Fig. 1C), and the IHC scores of SOX9 were decreased significantly from 5.8±2.7 (n=29) in normal cervical tissues to 4.1±3.0 (n=29) in cervical carcinoma *in situ* and to 2.4±2.1 (n=39) in cervical invasive carcinoma (P<0.05 between normal cervical tissues versus cervical carcinoma *in situ*; *P* < 0.01 between normal cervical tissues versus cervical carcinoma *P* < 0.05 between cervical carcinoma *in situ* versus cervical carcinoma). Additionally, we detected the expression of SOX9 protein in 8 normal cervical specimens and in 8 cervical carcinoma specimens by Western blot (Fig. 1D). The densitometry analysis showed that the average level of SOX9 in normal cervical tissue was significantly higher than that in cervical carcinoma (Fig. 1E, *P* < 0.05). All of these results consistently indicated that SOX9 expression is down-regulated in cervical carcinogenesis and supported the hypothesis that SOX9 might be a tumor suppressor in the development and progression of cervical carcinoma.

**Table 1:** SOX9 Expression in Different Tissue Specimens

Specimens	Tatol	SOX9 Staining		*P*
		Negative, No.(%)	Positve, No.(%)	
Normal	29	5 (17.2%)	24 (82.8%)	
CIN	29	12 (41.4%)	17 (58.6%)	< 0.05^a^
SCC	39	26 (66.7%)	13 (33.3%)	< 0.01^b^, < 0.05^c^

a Normal cervix versus CIN

b Normal cervix versus SCC.

c CIN versus SCC.

### SOX9 suppresses the tumor formation of cervical carcinoma cells *in vivo*

**Figure 2:**
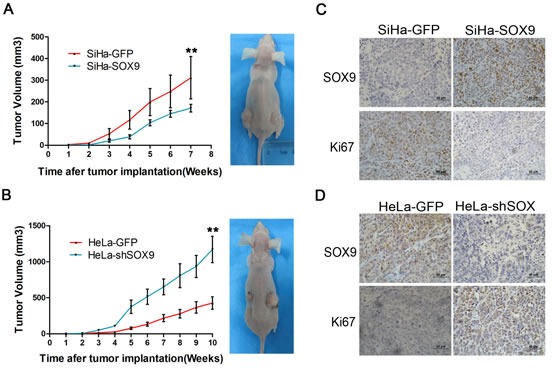
SOX9 suppresses cervical carcinoma tumorigenesis *in vivo* Tumor growth curves (left) and tumor formation (right) of the BALB/c nude mice are shown with SiHa-SOX9 cells (**A**), HeLa-shSOX9 cells (**B**) and with SiHa-GFP cells (A) and HeLa-shGFP cells (B) as controls. All of the groups were injected the same number of cells (1×10^5^ cells). The error bars indicate the mean tumor volume±SD for three independent experiments.*P < 0.05. SOX9 and Ki-67 were detected in SiHa (**C**) and HeLa (**D**) and in tumor xenografts by immunohistochemistry. Original magnifications, x400.

To explore the effect of SOX9 in tumor formation, xenograft assays were performed in BALB/c nude mice. The volumes of the solid tumors were monitored, and then tumor growth curves were graphed. Although the same number of cells was implanted (1×10^5^ cells), the tumor formation rate of the SOX9-overexpressing cells (SiHa-SOX9 cells) was much lower than that of the control cells (SiHa-GFP cells) (*p* < 0.01; Fig. 2A). Furthermore, the tumor xenografts formed by HeLa-shSOX9 cells grew much faster than tumors formed by the control cells (HeLa-shGFP cells) (*p* <0.01, Fig. 2C) because HeLa cells express high levels of endogenous SOX9 protein.

To further explore the relationship between SOX9 expression and cell growth in the tumor xenografts, paraffin sections of all tumor xenografts that were formed by SiHa-SOX9, SiHa-GFP, HeLa-shSOX9 and HeLa-shGFP cells were stained simultaneously with anti-Ki-67 and anti-SOX9 antibodies by immunohistochemistry. The tumors formed by SiHa-SOX9 cells were stained much more strongly for SOX9 but exhibited much weaker staining for Ki-67 than those formed by SiHa-GFP cells (Fig. 2B). Similarly, the tumors formed by HeLa-shGFP cells were stained much more strongly for SOX9 but were stained much more weakly for Ki-67 than tumors formed by HeLa-shSOX9 cells (Fig. 2D). All of these results demonstrated that the expression of Ki67 was inversely correlated with the expression of SOX9 in these xenograft cervical carcinomas, which implies that that SOX9 suppresses the formation of cervical carcinomas through the inhibition of cell proliferation.

### SOX9 inhibits the proliferation of cervical carcinoma cells *in vitro*

**Figure 3:**
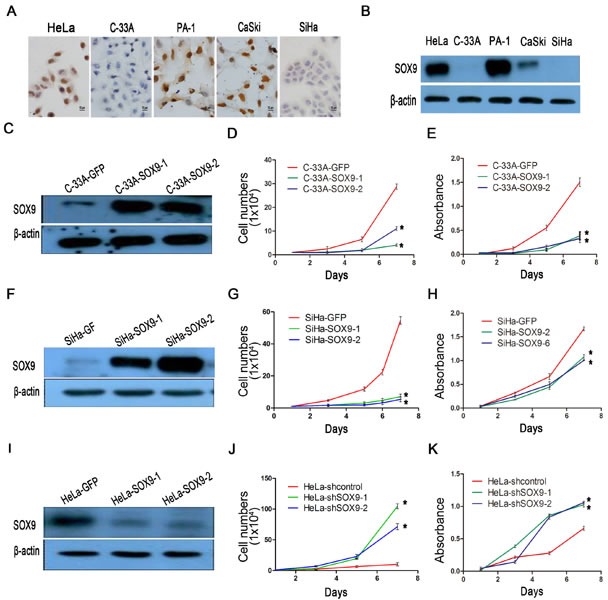
SOX9 inhibits the proliferation of cervical carcinoma cells *in vitro* **A.** Immunocytochemistry for SOX9 protein is shown in cervical cancer cell lines. Original magnification, x1000. **B.** The protein level of SOX9 in cervical cancer cells was analyzed by Western blot. The stably transfected cells were identified to detect the expression of SOX9 in C33A-GFP, C33A-SOX9-1,2**C.**, SiHa-GFP, SiHa-SOX9-1,2**F.** HeLa-shControl, and HeLa-shSOX9-1,2**I.**. β-actin was used as the loading control for the Western blot. The cell proliferation was determined by both growth curves and MTT assay in C33A-GFP, C33A-SOX9-1,2**D,E.**, SiHa-GFP, SiHa-SOX9-1,2(G,H), HeLa-shControl and HeLa-shSOX9-1,2**J,K.**. Data show the mean±SD from three independent experiments.*P <0 .05. **P <0 .01.

We also detected the expression of SOX9 in cervical carcinoma cell lines by immunocytochemistry (Fig. 3A) and Western blot (Fig. 3B). The results showed that the level of SOX9 expression was high in HeLa and CaSki cells, but was low in SiHa and C33A cells; the PA-1 cell line served as the positive control. Therefore, we overexpressed exogenous SOX9 in C33A and SiHa cells by stable gene transfection (Fig. 3C and 3F). We also knocked down SOX9 in HeLa cells by small interfering RNA (Fig. 3I) to determine whether SOX9 affected the functions of cervical carcinoma cells. The SOX9-expressing SiHa (SiHa-SOX9-1 and -2) and C33A cells (C33A-SOX9-1 and -2) had a significantly reduced capacity for proliferation than their respective controls (C33A-GFP, SiHa-GFP), as measured by growth curves(Fig. 3D and 3E, *p* < 0.05) and MTT assay (Fig. 3G and 3H, *p* < 0.05). Meanwhile, HeLa cells in which SOX9 was knocked down (HeLa-shSOX9-1 and -2) by siRNA showed a significantly higher capacity for proliferation than the control cells (HeLa-shGFP) (Fig. 3J and 3K; *p* < 0.05). These data demonstrated that the expression of SOX9 inhibits proliferation of cervical carcinoma cells *in vitro*.

### SOX9 inhibits cell proliferation through blocking the G1/S phase cell cycle transition in cervical carcinoma cells

**Figure 4:**
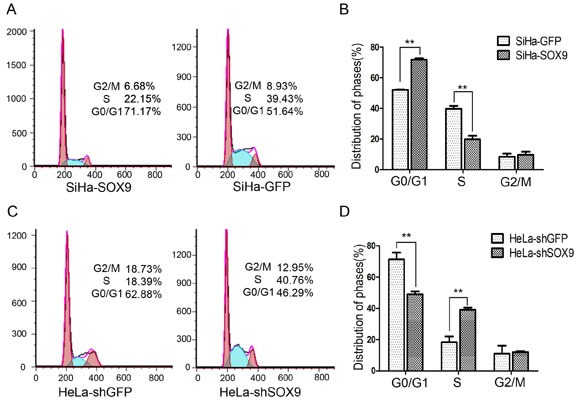
SOX9 inhibits the proliferation of cervical carcinoma cells by blocking G1/S phase transition **A.** A cell cycle analysis of SiHa-GFP and SiHa-SOX9 was performed by fluorescence-activated cell sorting (FACS). **B.** The percentage of cells that were distributed among the three phases are shown with histograms. **C.** A cell cycle analysis of HeLa-shGFP and HeLa-shSOX9 was performed by fluorescence-activated cell sorting (FACS). **D.** The percentage of cells that were distributed among the three phases are shown with histograms. Data are the mean±SD from three independent experiments.*P < 0 .05. **p < 0.01.

Cell cycle analysis by fluorescence-activated cell sorting (FACS) was performed to identify how SOX9 affects cell proliferation. As shown in Fig. 4A and 4B, the proportion of SiHa-SOX9 cells in G0/G1 phase (71.17%) was significantly higher than that of SiHa-GFP cells (51.64%), and the proportion of SiHa-SOX9 in S phase (22.15%) was significantly lower than that of SiHa-GFP cells (39.43%, *P* < 0.05). The ratio of SiHa-SOX9 cells in G1/S phase (71.17%/22.15%, 3.21) was much higher than that of SiHa-GFP cells (51.64%/39.43%, 1.31), which suggests that the expression of SOX9 caused SiHa cells to arrest at the G1/S phase transition point. Furthermore, the ratio of HeLa SOX9-knockdown cells in G1/S (HeLa-shSOX9, 46.29%/40.76%,1.14) was much lower than that of the control cells (HeLa-shGFP, 62.88%/18.39%, 3.42, Fig. 4C and 4D), which indicates that the knockdown of SOX9 induced the HeLa cells in G1/G0 to enter S phase. Thus SOX9 inhibits cell proliferation in cervical carcinoma cells at the G1/S phase transitionwhether the cervical carcinoma cells express SOX9 protein.

### SOX9 upregulated the expression of p21 in cervical cancer cells and cervical carcinoma tissues of patients

**Figure 5:**
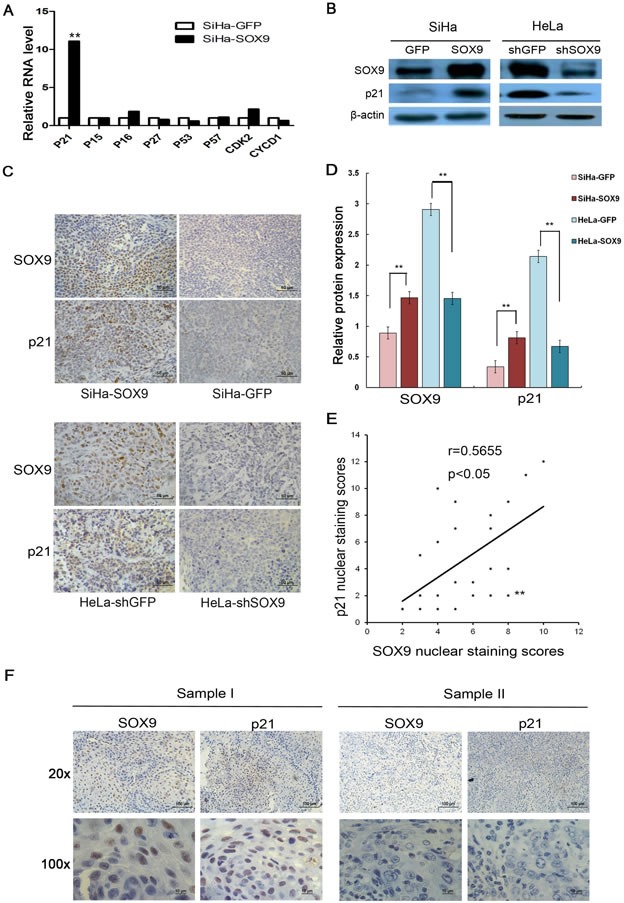
SOX9 transactivated p21 expression in cervical cancer cells and in cervical carcinoma tissues of patients **A.** Real-time PCR analysis is shown for the mRNA levels of various cell cycle regulatory genes in SiHa-SOX9 and SiHa-GFP cells. **B.** SOX9 and p21 protein expression was measured by Western blot in SOX9-overexpressing cells and cells where SOX9 was silenced. **D.**The densitometry analysis of SOX9 and p21 protein expression are shown in the SOX9-modified SiHa and HeLa cells( **p < 0.01). **C.** P21 was expressed in the nuclei of HeLa and SiHa xenografts. SiHa-GFP and HeLa-shGFP were used as controls. **F.**The expression of SOX9 and p21 was examined via immunohistochemical staining of 23 CC specimens. Upper, original magnifications, x200; Lower, original magnifications, x1000. **E.**The correlation of the staining of SOX9 and p21 was determined by Pearson's correlation test (r = 0.5655; *P* < 0.05). All the data are the mean±SD from three independent experiments.*P < 0 .05. **p < 0.01.

To investigate how SOX9 affects G1/S transition, a number of cell cycle regulatory factors, including p15, p16, p21, p27, p53, CDK2 and cyclin D1, were detected by real-time PCR in SiHa-SOX9 and SiHa-GFP cells. As shown in Fig. 5A, only the cyclin-dependent kinase inhibitor p21 was found to be much more highly expressed in SiHa-SOX9 cells compared with SiHa-GFP cells, which implies that p21 is a possible positively regulated target of SOX9. A Western Blot assay and a densitometry analysis were used to identify the relationship between SOX9 and p21 in both the SOX9-modified SiHa and HeLa cells. As shown in Fig. 5B and 5D, p21 protein was expressed more strongly in higher SOX9-expressing cells (SiHa-SOX9 and HeLa-shGFP) than in lower SOX9-expressing cells (SiHa-GFP and HeLa-shSOX9) (*P* < 0.01). The expression of p21 protein was consistent with the expression of SOX9 according to an immunohistochemical analysis of the tumor xenografts (Fig. 5C). Furthermore, the expression levels of p21 protein were also positively correlated with the expression levels of SOX9 protein in the panel of 23 CC samples (Fig. 5E and 5F, r=0.5655, *P* < 0.05). These data suggested that SOX9 possibly inhibits proliferation of cervical cancer cells through the up-regulation of p21.

### SOX9 transactivated the expression of p21 in cervical cancer through binding to the promoter of p21 *in vivo*

**Figure 6:**
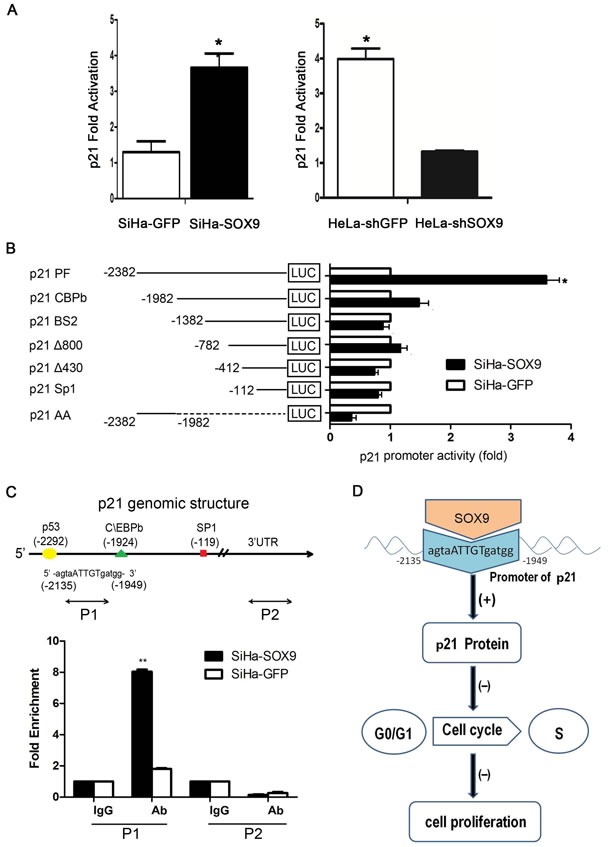
SOX9 transactivated the expression of p21 by binding to the Promoter of p21 *in vivo* **A.** Duplicate luciferase reporter assays were performed with the p21 promoter constructs in SOX9-modified SiHa and HeLa cells(*p < 0.05). **B.** The activity of a series of p21 promoter 5‘ deletion mutants was measured by a dual luciferase assay and presented as the fold change of the relative luciferase activity ratio of SiHa-SOX9 versus SiHa-GFP(*p < 0.05). **C.** Schematic structure of the p21 promoter is shown, including the locations and sequences of putative SOX9-binding sites and the quantitative chromatin immunoprecipitation (qChIP) primers that flank the regulatory region. The qChIP assay is shown in the p21 promoter region in SOX9-overexpressing SiHa cells or after transfection with empty vector. Immunoglobulin G (IgG) was used as the negative control. The binding activity is presented as the percentage of the total input. All the data are the mean±SD from three independent experiments (*P < 0.05,**P < 0.01). **D.** Schematic representation of the functional roles of Sox9 and its up-regulation of p21 protein expression through direct binding to the promoter of p21; this induces cell cycle arrest in the G1/S phase and the inhibition of the proliferation of cervical carcinoma.

To investigate the possible mechanism by which SOX9 up-regulated the expression of p21 in cervical cancer, the luciferase reporter assay was performed in duplicate by transient transfection with the p21 promoter constructs. As shown in Fig. 6A, the luciferase activity in SiHa-SOX9 cells was more than three times higher than that in SiHa-GFP cells (*P* < 0.05). Similarly, the luciferase activity in HeLa-shSOX9 cells was more than three times less than that in HeLa-shcontrol cells (*p* < 0.05). These results indicated that SOX9 transactivates the expression of p21 in cervical cancer cells.

To identify the binding sites of SOX9 in the p21 promoter, additional luciferase reporter assays were performed with the different deletion fragments of the p21 promoter. As shown in Fig. 6B, the luciferase activity of the full-length p21 promoter was significantly higher in SiHa-SOX9 cells than in SiHa-GFP cells (*p* < 0.05). The luciferase activity with different deletions in the p21 promoter was not significantly different in SiHa-SOX9 cells compared with SiHa-GFP cells, which suggested that the sequence between the nucleotides −2382 and −1982 in the p21 promoter may contain the SOX9 binding site. However, the luciferase activity with the only sequence between −2382 and −1982 was not shown to be significantly different between SiHa-SOX9 and the SiHa-GFP cells, which implied that the region between the nucleotides −2382 and −1982 might contain the binding sites for the transcriptional activation of p21 by SOX9. However, the other sequences in the p21 promoter were also necessary for transactivation of p21 by SOX9.

An experimentally proven binding site of SOX9, the consensus binding sequence 5′-agtgATTGTgatgg-3′, was found between −2382 and −1982 in the p21 promoter by information retrieved by the TRANSFAC^®^ database. Therefore, a qChIP assay was performed to identify whether SOX9 could bind to the 5′-agtgATTGTgatgg-3′ sequence between −2382 and −1982 in the p21 promoter in SiHa-SOX9 cells *in vivo*. After precipitation, real-time PCR with the specific P1 and P2 primers was designed and performed to amplify the p21 promoter binding region between −2135 and −1949, and the 3′-untranslated region(as a control). Real-time PCR revealed that the use of the P1 primers after precipitation with a SOX9 antibody led to an amplification that was four times greater in SiHa-SOX9 cells than in SiHa-GFP cells. Moreover, an amplification greater than 8-fold was observed compared with PCR after the precipitation of IgG in both cell types, or compared with that by the P2 primer (Fig. 6C, *P* < 0.01), which suggests that SOX9 could physically bind to the p21 promoter to function in transactivation. Thus, SOX9 transactivates p21 through direct binding to the promoter of p21.

## DISCUSSION

Here we showed that SOX9 is down-regulated in cervical cancer and represents tumor-suppressor in this malignancy. In contrast, SOX9 may serve as oncogene in lung, skin, prostate and brain cancers [12, 19-21]. In line with our results in cervical cancer, SOX9 has been shown to inhibit melanomas and endometrial tumors [15, 22]. It was previously reported that methylation levels in the SOX9 promoter in 156 cervical scrapings of normal tissues and different cervical lesions increased with the severity of cervical squamous lesions. In the present study, SOX9 was highly expressed in the normal cervix, but gradually decreased in cervical carcinoma *in situ*, as well as in invasive cervical carcinomas according to semiquantitative IHC and Western blot analysis (Fig. 1). Thus, the results of our SOX9 expression study in cervical carcinogenesis are consistent with those of the study by Wu et al. [18], which suggests that SOX9 may function as a tumor suppressor gene in the development and progression of cervical carcinomas.

To further explore the function of SOX9 in cervical carcinogenesis, xenograft assays, growth curves and MTT assay were performed in the SOX9-modified SiHa, HeLa and C33A cell lines. In all of these cell types, the up-regulation of SOX9 protein inhibited cell proliferation and tumor formation, while the down-regulation of SOX9 promoted cell proliferation and tumor formation (Fig. 2 and 3). These results supported the notion that SOX9 functions as a tumor suppressor in cervical carcinogenesis *in vivo* and *in vitro*. A similar SOX9 function had also been found in endometrial tumorigenesis [11, 16], which implies that SOX9 might function as a tumor suppressor in human genital tract malignancies.

Cell cycle analysis on the SOX9-modified SiHa, HeLa and C33A cells revealed that SOX9 causes G1 arrest. SOX9 regulates p14, p53, p21, CDK4, and cyclin D1 [12, 23, 24]. In our study, real-time PCR and Western blot analyses demonstrated that p21 is the only cell cycle protein that is positively regulated by SOX9 in all SOX9-modified cervical cancer cell lines, as well as in their respective tumor xenografts. Furthermore, SOX9 expression was positively correlated with nuclear p21 expression in 23 cervical specimens from patients with CC (r = 0.5655, *P* < 0.05), which supports the notion that SOX9 positively regulates p21 expression in cervical carcinogenesis. Also, SOX9 positively regulates p21 expression in melanoma and endometrial carcinoma cells [15, 16]. However, Jiang et al. has reported that p21 was negatively regulated by SOX9 in lung adenocarcinoma cell lines [12]. Therefore, although SOX9 regulates p21 expression in human malignancies, the outcome of this regulation is tissue-dependent [25]. In our study, a luciferase reporter assay and qChIP assay confirmed that SOX9 directly transactivated p21 through binding with the proximal agtgATTGTgatgg consensus sequence within the p21 promoter in cervical carcinoma cells (Fig. 6A-6C). A similar result was also found in endometrial carcinoma cells by Saegusa et al. [16]. We speculate that restoration of SOX9 is a potential strategy for the treatment of certain types of cervical carcinomas.

## MATERIALS AND METHODS

### Human tissue samples and immunohistochemistry

A total of 29 normal cervical tissues(NC), 29 cervical carcinomas *in situ*(CIN) and 39 cervical carcinoma(CC) tissues were obtained from volunteer patients who were hospitalized from January 2008 to December 2013 at the First Affiliated Hospital of Xi'an Jiaotong University. Eight normal cervical tissues and eight fresh invasive cervical carcinoma tissues were also collected from the First Affiliated Hospital of Xi'an Jiaotong University for Western blot analysis. None of the patients had received chemotherapy, immunotherapy or radiotherapy before collection. The specimens that were obtained for routine pathological studies were fixed in 10% buffered formalin, embedded in paraffin and stained with a rabbit polyclonal antibody against human SOX9(1:500 dilution; sc-20691; Santa Cruz Biotechnology, Santa Cruz, Calif, USA). A positive expression was defined as a reddish brown precipitate observed in the nuclei. All slides were examined under an Olympus-CX31 microscope (Olympus, Tokyo, Japan), and two investigators scored the results in ten randomly selected fields at ×400 magnification. The SOX9 staining was classified into 2 categories: negative expression or positive expression based on the percentage of positive cells and the staining intensity [26, 27]. The percentage of positive cells was divided into 5 degrees of scores: 0 (0%); 1 (1%–10%); 2 (11%–50%); and 3 (>50%). The intensity was divided into 4 degrees of scores: 0 (negative); 1 (weak); 2 (moderate); and 3 (strong). The final immunoreactivity scores (IRS) for each case were determined by the product of the percentage score and the intensity score. The scores were based on a median of the value of the IRS; final scores that were less than 3(not including 3) were defined as negative, while final scores of more than 3(including 3)were defined as positive. Stromal cells were used as a negative control.

### Cell culture and immunocytochemistry

The human cervical cancer cell lines HeLa, SiHa, CaSki, C33-A and the human teratoma cell line PA-1 were purchased from the American Type Culture Collection (Manassas, VA) and cultured according to their specifications at 37°C in a humidified incubator with 5% CO_2_. To detect the expression of SOX9 in the cell lines, immunocytochemistry was performed after the cells were seeded on cover slips for 48 hours, fixed with 4% paraformaldehyde for 20 minutes and permeabilized with 0.2% Triton X-100 for 20 minutes at room temperature.

### Cell proliferation assay, 3-(4,5-dimethylthiazole-2-yl)-2,5-diphenyl tetrazolium bromide

Cervical cancer cells were seeded at a density of 5×10^4^ cells per well in triplicate in 6-well plates. The cell numbers were counted every day for 1 week with a hemocytometer, and the data are from three independent experiments. Cell growth curves were used to determine the rate of cell proliferation. Cell viability was assessed with 3-(4,5-dimethylthiazole-yl)-2,5-diphenyl tetrazolium bromide ((MTT), Sigma-Aldrich) dye according to a standard protocol. Briefly, the cells (1000 cells per well) were seeded in 96-well plates; six parallel samples were used for each condition. A total of 20 μl of MTT (5 mg/ml) was added to each well followed by incubation for 4 hours at 37°C; the cells were then dissolved in 150 μl of dimethyl sulfoxide. The number of live cells was determined by a measurment of the absorbance at 490 nm (Bio-Rad).

### Immunoblot assay

Western blot analyses were performed [28] with the lysates from fresh tissues and cells as previously described. The membranes were incubated with rabbit polyclonal antibodies against human SOX9 and p21 (1:500 dilution, sc-528, Santa Cruz Biotechnology) as well as with the mouse monoclonal anti-β-actin (1:500 dilution, sc-47778, Santa Cruz Biotechnology) at 40°C overnight. This was followed by an incubation with the secondary antibody, which was either horseradish peroxidase-conjugated antirabbit or antimouse immunoglobulin G (IgG; Thermo Fisher Scientific, New York, NY). Proteins were visualized with the Immobilon Western Chemiluminescent HRP Substrate (Millipore, Billerica, MA) on x-ray film. Densitometry measurements of the scanned bands were performed with AlphaView SA software (Cell Biosciences, Santa Clara, CA). Data were normalized to β-actin for quantification purposes.

### Vector construction and transfection

Full-length SOX9 cDNA was amplified with the following primers: forward, 5′- CCGGAATTCGATGAATCTCCTGGA-3′ and reverse, 5′-CGGATCCGTCAAGGTCGAGTGAG-3′. The SOX9 DNA fragment was subsequently cloned into the EcoRI and BamHI (TaKaRa, Tokyo, Japan) sites of the internal ribosome entry site vector pIRES-AcGFP (Clontech, Mountain View, CA) to generate the pIRES-AcGFP-SOX9 recombinant plasmid. SOX9 pGPU6/GFP/Neo siRNA was used to generate a negative control plasmid as well as plastmids that express SOX9-specific short hairpin RNA (shRNA). All transfection experiments were performed with Lipofectamine 2000 reagent (Invitrogen, Carlsbad, CA) according to the manufacturer's instructions, and stable clones from HeLa, C33A and SiHa cells were each selected with 1 mg/ml G418 (Calbiochem, La Jolla, CA).

### Flow cytometry

The cell cycle analysis was performed by ﬂow cytometry (FACS; Becton Dickinson, Franklin Lakes, NJ). Approximately 1×10^6^ cells were collected and fixed in 70% ethanol at 4°C overnight. Then, the cells were treated with 1 mg/ml RNase A and stained with 20 μg/ml propidium iodide (Sigma-Aldrich) thirty minutes before FACS. The cell cycle distribution was analyzed with Mod-Fit LT software.

### PCR analysis

Total RNA from cultured cervical carcinoma cells was extracted with TRIzol Reagent (Invitrogen) and reverse transcribed with the RevertAid™ First Strand cDNA Synthesis Kit (#K1621; Fermentas, Burlington, Ontario, Canada) with GAPDH as the internal control. PCR products were separated on a 2.5% agarose gel and were observed with the Molecular Imager Gel Doc™ XR+ system (Bio-Rad, Hercules, CA). Total cDNA was used as a template for PCR amplification of SOX9. Real-time quantitative PCR was performed in triplicate for each primer set and for each cell sample in an iQ5 multicolor real-time PCR Detection System (Bio-Rad, Hercules, Calif) with SYBR Premix Ex Taq™ II (TaKaRa); the reults were analyzed with Bio-Rad IQ5 software v. 2.0 according to the manufacturer's instructions. All experiments contained at least biological duplicates and assay triplicates; the results were analyzed via the 2-ΔΔCt method using GAPDH and β-actin as the housekeeping genes. The protocol for realtime PCR was 1 cycle of 95°C for 30 seconds, 40 cycles of 95°C for 5 seconds, and 60°C for 30 seconds followed by a dissociation stage. The cycle threshold value was determined as the point at which the fluorescence exceeded a preset limit determined by the instrument's software. The primers for β-actin were adapted from a previous study [29].

### Tumor xenograft assay

Cells in the exponential growth phase were harvested for inoculation. Tumor cells (1×10^5^or 1×10^4^) were injected into the subcutis on the dorsum of 4- to 6-week-old female BALB/c-nude mice(Jackson Laboratories, Inc., Bar Harbor, ME). Mice(6 mice per group) were monitored weekly for body weight and tumor size for up to 11 weeks after the injection. The tumor volume (V) was determined by the length (a) and width (b) as V= ab^2^/2 [27]. The experimental protocols were evaluated and approved by the Animal Care and Use Committee of the Medical School of Xi'an Jiaotong University. The mice were sacrified, and the tumors were fixed in 4% paraformaldehyde (pH 7.0) and paraffin- embedded for histological analysis.

### Dual luciferase reporter assay

Briefly, plasmids containing firefly luciferase reporters were co-transfected into tumor cells in triplicate using Lipofectamine 2000 (Invitrogen). PMiniTK-RL was used as an internal control. After 48 hours, the cell monolayers were washed with PBS, harvested by scraping and resuspended in passive lysis buffer. The luciferase activity was measured by a luminometer (Promega, Madison, WI), and the transfection efficiency was normalized to the paired Renilla luciferase activity with the Dual Luciferase Reporter Assay system (Promega) according to the manufacturer's instructions. The specific promoter activity was expressed as the fold change of the experimental group versus the control group.

### Quantitative chromatin immunoprecipitation

Quantitative chromatin immunoprecipitation (qChIP) assays were performed as previously described [30] with the EZ-ChIPTM Assay Kit (Cat#17–371; Millipore). For quantitative ChIP analysis, the cells were lysed and sonicated to produce DNA fragments of 0.2–1 kb. Chromatin protein complexes were immunoprecipitated with anti-SOX9 antibody (sc-130911; Santa Cruz Biotechnology). The negative control was immunoprecipitated with normal mouse IgG, and input DNA was precipitated without antibody. Regions of interest were amplified from precipitated samples by real-time polymerase chain reaction (PCR). The p21 promoter, which contains an experimentally proven binding site for SOX9, was amplified with the following primer pair (designated as P1): P1F, 5′- GATTCTCCCACCTCTGCC-3′ and P1R,5′- TGTTTGCCCTGAGTCCTG-3′. The 3′untranslated region of the p21 gene was amplified as a control for the qChIP assay using the following primer set (designated as P2): P2F, 5′-GCCCGCTCTACATCTTCT-3′ and P2R, 5′-AAATGCCCAGCACTCTTA-3′. Each sample was assayed in triplicate, and the amount of precipitated DNA was calculated as a percentage of the input sample [31].

### Statistical analysis

Statistical analysis was performed with SPSS 15.0 software (SPSS Inc., Chicago, IL). All data were expressed as the group means ± standard deviation of the mean (SD). The 2-tailed Chi-square test or Fisher's exact test was used to determine the significance of the differences between the covariates. A univariate analysis was analyzed by Student's *t*-test (two-tailed) and the Mann-Whitney U-test. The correlation of the expression of SOX9 and the expression of p21 in CC was analyzed by Pearson's correlation test. In all tests, *P* < 0.05 was defined as statistically significant.
